# Wm. Brown Gildea

**Published:** 1846-09

**Authors:** 


*Obituary.*—It will be seen by the following obituary, which we copy from a St. Louis paper, that another of the members of the American Society of Dental Surgeons has deceased:

"Died, at Fort Helvetia, (Capt. Sutter's,) Upper California, on the 8th of February, 1846, in the 26th year of his age, Wm. Brown Gildea, M.D., Dental Surgeon, late of St. Louis, Mo.

"He was born in Dauphin county, Pennsylvania, and became a resident of this city about eight years ago, when he entered upon the study of medicine and dental surgery under the instruction of his uncle, Dr. B. B. Brown. In the spring of 1843 he graduated at the St. Louis University. In May, 1845, he accompanied the emigrant expedition to California, with a view of practising medicine in that country.

"Modest, to a fault, in his deportment, retiring in his intercourse with society, and with a disposition at once affable and generous, he won the respect and esteem of his acquaintances, and the warm friendship of those who intimately knew him. A laborious student, he acquired an excellent knowledge of his profession, as well as of those branches of science which directly related thereto. Enterprising and energetic, the adventurous life incident to a partially known region had a charm for him that no inducement could break.

" But far from the home of his youth, and the endearing scenes of his manhood—in a strange land, amid strangers and wanderers, like himself, he has found the stranger's last resting place—the grave—where friendship may never shed a tear on his lonely tomb, nor the hand of affection strew flowers upon the green sod that covers his dust—where kindred cannot renew the quickly mouldering traces of the solitary sleeper, nor benevolence breath a prayer over the urn of the departed. Soon shall the spot of his sepulture be known no more forever; but his place in the heart of those who loved him, and who knew his many virtues, will long be cherished, as affection's best monument to the mourned and lost."

